# Data-driven frailty and reserve phenotypes in older outpatients: a cluster analysis of Comprehensive Geriatric Assessment

**DOI:** 10.3389/fragi.2025.1678407

**Published:** 2026-01-12

**Authors:** Maristella Belfiori, Francesco Salis, Benedetta Puxeddu, Martina Mulas, Monica Puligheddu, Antonella Mandas

**Affiliations:** 1 Department of Medical Sciences and Public Health, University of Cagliari, Cagliari, Italy; 2 Department of Biomedical Sciences, University of Cagliari, Cagliari, Italy

**Keywords:** aging, cluster analysis, comprehensive geriatric assessment, frailty, reserve capacity

## Abstract

**Background:**

The progressive aging of the population represents a critical public health challenge. Within this context, the management of frailty has emerged as a central priority in geriatric care, with Comprehensive Geriatric Assessment (CGA) widely recognized as the gold-standard tool for its evaluation. This study aimed to stratify a large cohort of older adults using a multidimensional approach based on CGA, employing Principal Component Analysis (PCA) and Cluster Analysis to identify distinct phenotypic profiles.

**Materials and methods:**

A cross-sectional study was conducted on 1055 outpatients aged ≥65 years, assessed at the Geriatric Outpatient Service of the University of Cagliari between 2020 and 2024. All participants underwent a CGA. PCA was performed on selected CGA variables, and the resulting components were used for a hierarchical cluster analysis. Post-hoc comparisons between clusters were conducted using ANOVA, Chi-squared or Fisher tests, as appropriate.

**Results:**

PCA identified four principal components explaining 73.5% of total variance. The first component represented a Frailty Axis, while the second reflected Reserve Capacity. Cluster analysis based on these two axes revealed four distinct phenotypes: (I) Vulnerable Low-Complexity (younger patients with low comorbidity but significant cognitive, functional and nutritional impairments), (II) Resilient High-Reserve (low comorbidity with preserved cognitive, functional, and nutritional status and high educational attainment), (III) Resilient Frailty (high comorbidity, functional and nutritional deficits but preserved cognitive reserve) and (IV) Globally Frail (older patients with high comorbidity with multidomain impairments).

**Conclusion:**

These findings demonstrate the ability of CGA, combined with PCA-informed clustering, to identify clinically meaningful frailty and resilience patterns in older adults. The study highlights the role of educational attainment as a key factor contributing to clinical reserve; conversely, it showed that demographic characteristics, laboratory markers, and comorbidities align with frailty.

## 1 Introduction

Population aging in Italy is a rapidly intensifying demographic phenomenon. As of January 2025, data from the Italian National Institute of Statistics (ISTAT) indicate that approximately 15 million individuals - 24.7% of the population - are aged 65 years or older, representing a 0.4 percentage point increase compared to 2024. Additionally, 4.1% of the population is 85 years or older, placing Italy among the countries with the highest proportion of older adults globally (Italian National Institute of Statistics ISTAT, 2025). These demographic trends are anticipated to accelerate in the coming decades, with projections suggesting that over 34% of Italy’s population will be aged 65 or older by 2070 (Italian National Institute of Statistics ISTAT, 2021). This trend aligns with global patterns, as the population aged 65 and above is expected to exceed 1.5 billion by 2050 (United Nations et al., 2020).

Such demographic changes carry substantial implications for the healthcare system. The growing prevalence of chronic diseases and complex care needs among older adults is leading to a marked increase in demand for hospitalizations, outpatient services, and long-term care. In this context, the challenge is not merely to extend lifespan, but to enhance health span -the period of life spent in good health and functional autonomy-though more targeted and effective strategies. A key issue in this field is the marked clinical heterogeneity observed among older adults (Cesari et al., 2018; Hoogendijk et al., 2019). While some maintain independence and good health, others face progressive functional decline, multimorbidity, and increased social vulnerability. Notably, 28.7% of individuals in Italy aged 65 and older report experiencing severe limitations in their daily activities (Frova et al., 2024). Relying solely on chronological age to guide healthcare decisions fails to capture the full diversity of individual health needs, leading to both over-treatment and under-treatment. This underscores the pressing need for meaningful clinical tools, capable of assessing individual needs and supporting multidimensional approaches to stratification and care planning. Central to this complexity is the concept of frailty, a multidimensional syndrome characterized by decreased physiological reserves and increased vulnerability to stressors (Clegg et al., 2013). Frailty is well recognized as a predictor of negative outcomes, including disability, hospitalization, institutionalization, and mortality. Additionally, it is associated with higher healthcare utilization and costs (Clegg et al., 2013; Veronese et al., 2017), as frail older adults require more complex and resource-intensive care. Therefore, identifying and properly managing frailty has become a central priority in both geriatric care and public health planning.

In recent years, the importance of early frailty identification has been increasingly emphasized by international guidelines, including those issued by the World Health Organization (WHO) (World Health Organization, 2017). The WHO advocates for a transition from reactive to proactive, prevention-focused care models, such as the Integrated Care for Older People approach (World Health Organization, 2019). This shift underscores the need for stratification strategies that move beyond simple one-dimensional scores or diagnostic categories, favouring instead multidimensional assessments capable of capturing the complexity of aging trajectories.

To address this, the implementation of a Comprehensive Geriatric Assessment (CGA) has been widely recognized as the gold-standard multidimensional tool to evaluate the medical, psychological, functional, and social domains of older adults (Rubenstein et al., 1991; Brigg et al., 2022). Evidence suggests that CGA, particularly when implemented in specialized geriatric settings, is associated with improved outcomes, including a increased likelihood of being alive and living at home following hospital discharge (Brigg et al., 2022).

This study aims to stratify a large outpatient geriatric cohort using a multidimensional approach based on Principal Component Analysis (PCA) and Cluster Analysis, to identify distinct clinical phenotypes and characterize the underlying patterns of frailty.

## 2 Materials and methods

The study comprised 1055 outpatients from the Geriatric Outpatient Service at the University of Cagliari, evaluated between January 2020 to December 2024. Participants were individuals aged 65 years and older who underwent a CGA. Patients without informed consent or lacking anamnestic data or a CGA were excluded from the study.

### 2.1 Clinical and laboratory assessment

Trained geriatricians collected demographic data, including sex and years of education. Information regarding current or past smoking habits and current or past alcohol consumption was also recorded. Laboratory analyses included Red Blood Cell Count (RBCs), Haemoglobin (Hb), White Blood Cell Count (WBCs), Platelet Count (PLT), Serum Glucose, Serum Creatinine and Blood Urea Nitrogen (BUN) as well as Estimated Glomerular Filtration Rate (eGFR), calculated using the Chronic Kidney Disease Epidemiology Collaboration (CKD-EPI) 2021 equation. Additional parameters included Total Protein, Albumin, Total Cholesterol, High-Density Lipoprotein Cholesterol (HDL-C), Triglycerides (TG), and Low-Density Lipoprotein Cholesterol (LDL-C), the latter estimated via the Friedewald formula. Levels of Vitamins B_12_, B_9_, and D, Parathyroid hormone (PTH), and Fibrinogen (FBG) were also measured.

### 2.2 Comprehensive Geriatric Assessment

Participants underwent a CGA, which included evaluations of cognitive domain, mood, functional ability, mobility and nutritional status. Cognitive function was assessed using the Mini-Mental State Examination (MMSE) (Lancu and Olmer, 2006; Magni et al., 1996), which evaluates orientation, memory, attention, language, and visual construction abilities. A score below 24 typically indicates cognitive impairment. Mood was assessed using the 15- item Geriatric Depression Scale (GDS) (Belfiori et al., 2024; Yesavage et al., 1982), which scores ranging from 0 (no depressive symptoms) to 15 (severe depressive symptoms);. Scores above 5 suggest the presence of depressive symptoms.

Functional and mobility domains were evaluated using the following tools:

- Activities of Daily Living (ADLs) scale (Hyejin et al., 2021; Wade et al., 1988), which assesses the ability to perform basic daily activities. Scores range from 0 (complete dependence) to 100 (full independence).
- Instrumental Activities of Daily Living (IADLs) scale (Hyejin et al., 2021; Graf, 2008), which evaluates the ability to carry out daily tasks that support their life at home and in the community. Scores range from 0 (complete dependence), to 8 (full autonomy).
- Performance-Based Physical Test (PPT) (Hegedus and Cook, 2015; Reuben and Siu, 1990), an objective measure of physical performance based simulated daily activities. A score >20 indicates absence of disability; scores between 11 and 20 represents moderate disability; and scores ≤10 reflect severe disability.
- Tinetti Performance-Oriented Mobility Assessment (POMA) (Singh et al., 2024; Yang et al., 2023), a widely used tool for evaluating balance and gait in older adults. Scores range from 0 to 28, with values <25 indicating a risk of falls.

Nutritional status was assessed using the Mini Nutritional Assessment (MNA) (Calvo et al., 2012; Soini et al., 2004) with scores below 24 indicating malnutrition risk. The assessment incorporates Body Mass Index (BMI) (Volkert et al., 2019), whose thresholds for older adults differ from those applied to younger populations. According to ESPEN guidelines, a BMI value below 22 kg/m^2^ is considered underweight in older individuals.

The Exton-Smith Scale (ESS) (Norton et al., 1962) was used to evaluate the risk of developing pressure sores. This tool consists of a structured five-item questionnaire, with score below 13 indicating a higher risk of pressure lesions.

To classify comorbidities, we used the Cumulative Illness Rating Scale (CIRS) (Salis et al., 2022), which identifies and categorizes patients according to 14 specified medical conditions (sub-item): Cardiovascular diseases, Arterial Hypertension, Vascular diseases, Respiratory conditions, Ophthalmological and Otolaryngologic disorders, Gastrointestinal diseases, Hepatic conditions, Renal disorders, Genitourinary conditions, Musculoskeletal and Dermatological disorders, Central and Peripheral Nervous System pathologies (excluding dementia), Endocrine Metabolic Diseases and Psychiatric/Behavioral disorders (encompassing dementia, mood disorders, anxiety, agitation, delirium, and psychosis). The CIRS provides a total score (CIRS Tot.) and the Severity Index (CIRS ISC), calculated as the mean severity score across the first 13 items (CIRS ISC-13) and across all 14 itemsitems (CIRS ISC-14).

Additionally, we employed the PRISMA-7 questionnaire, a validated seven-item screening tool designed to identify frailty in older adults. It assesses mobility limitations, comorbidities, and the need for assistance in daily activities. A score ≥3 is generally indicative of an increased risk of frailty (Raîche et al., 2007).

### 2.3 Statistical analysis

Given the sufficiently large sample size (>100 patients), we assumed that the sampling distribution of the means approximates normality in accordance with the Central Limit Theorem. Consequently, quantitative variables are reported as mean and standard deviation (SD), while categorical variables as percentages.

A multivariate approach was employed to explore underlying patterns in the data collected through the CGA. Specifically, PCA was performed reduce dimensionality and synthesize the selected variables into common components, followed by Cluster Analysis to categorize patients based on their multivariate profiles. To ensure stability of component loadings and interpretability, a focused selection of meaningful and representative variables was included in the PCA, covering key CGA domains: cognitive function (MMSE), mood (GDS), functional status (ADLs, IADLs), nutritional status (MNA), and comorbidity burden (CIRS). For the latter, both CIRS tot. and CIRS ISC were used. Demographic factors, including age and years of education, as well as BMI -included for its clinical significance in identifying patterns of underweight or obesity beyond the scope of the MNA- were also assessed. Prior to PCA, all selected variables were standardized (mean = 0, SD = 1). Components with meaningful contribution were interpreted based on factor loadings, with loading ≥ |0.30| considered significant. PCA results were graphically represented using a biplot.

Subsequently, cluster analysis was performed using the principal components (PCs) derived from PCA as input. Euclidean distance was used as the similarity measure, and the Ward’s linkage method was applied. A dendrogram was constructed and visually inspected, with the optimal number of clusters defined using both graphical methods (dendrogram cut-off) and analytical approaches (silhouette method and Silhouette Score analysis). Clusters were visualized on the PCA map to examine their spatial distribution. Post-hoc pairwise comparisons among clusters were conducted for additional variables not included in the PCA, such as functional measures, comorbidity indicators, and laboratory markers, following a significant ANOVA, with Bonferroni correction. Differences in categorical variables across clusters were assessed using Chi-squared or Fisher’s exact tests, as appropriate.

All statistical analyses were performed using RStudio software (version 4.4.2) with a significance thresholdof p < 0.05. Further descriptive statistics and graphical representations related to the PCA, and clustering process are reported in the Supplementary Tables S1–S2; Supplementary Figure S1.

### 2.4 Institutional review board statement

The study was conducted in accordance with the Declaration of Helsinki and approved by the Ethics Committee of the University of Cagliari (protocol code NP/2022/1382, 30 March 2022).

## 3 Results

The demographic and clinical characteristics of the sample are detailed in Table 1. Notably, women comprised 69.1% of the cohort, and 59.23% had an education level of less than 5 years, including 3.5% of participants with no formal education.

**TABLE 1:** Characteristics of the sample.

Demographic data	
Age (years)	79.88 (7.01)
Male. n (%)	326 (30.9)
Education (years)	6.40 (3.85)
Primary or below. n (%)	628 (59.53)
Lower secondary school. n (%)	247 (23.41)
Upper secondary school or above. n (%)	180 (17.06)
Alcohol consumers	1.1.2
Current. n (%)	43 (4.1)
Former. n (%)	32 (3.0)
Smokers	1.1.3
Current. n (%)	73 (6.9)
Former. n (%)	297 (28.2)
BMI	28.40 (5.93)

CGA	
MMSE	21.94 (5.39)
GDS	6.75 (3.81)
ADLs	78.64 (18.63)
IADLs	3.63 (2.36)
PPT	14.24 (6.55)
POMA	17.01 (7.15)
MNA	22.08 (4.29)
ESS	15.28 (3.73)
CIRS Tot.	29.89 (4.71)
CIRS ISC-13	2.09 (0.34)
CIRS ISC-14	2.14 (0.33)

Laboratory assessment	
RBCs (x 10^3^/µL)	3.46 (12.58)
Hb (g/dL)	12.67 (1.74)
WBCs (x 10^3^/µL)	6.99 (2.80)
PLTs (x 10^3^/µL)	236.43 (74.96)
Serum glucose (mg/dL)	109.09 (34.33)
Serum creatinine (mg/dL)	1.03 (0.58)
BUN (mg/dL)	35.09 (23.68)
eGFR (mL/min/1.73 m^2^), CKD-EPI	63.25 (19.32)
Total protein (g/dL)	7.00 (0.59)
Albumin (g/dL)	3.89 (0.39)
Total Cholesterol (mg/dL)	191.54 (44.32)
HDL-C (mg/dL)	57.98 (15.92)
TG (mg/dL)	102.87 (46.64)
LDL-C (mg/dL)	116.48 (40.88)
Vitamin B_12_ (ng/mL)	363.87 (210.12)
Vitamin B_9_ (ng/mL)	6.95 (4.76)
Vitamin D (ng/mL)	27.30 (15.81)
PTH (pg/mL)	73.55 (48.51)
FBG (mg/dL)	377.96 (91.83)

Data are presented as n (%) or mean (SD). Abbreviations: ADLs, Activities of Daily Living; BUN, Blood Urea Nitrogen; BMI, Body Mass Index; CGA, Comprehensive Geriatric Assessment; CIRS Tot., Cumulative Illness Rating Scale Total Score; CIRS ISC, Cumulative Illness Rating Scale Severity Index; CKD-EPI, Chronic Kidney Disease Epidemiology Collaboration; eGFR, Estimated Glomerular Filtration Rate; ESS, Exton-Smith Scale; ENT, Ear, Nose, and Throat; FBG, Fibrinogen; GDS, Geriatric Depression Scale; Hb, Haemoglobin; HDL-C, High-density lipoprotein Cholesterol; IADLs, Instrumental Activities of Daily Living; LDL-C, Low-Density Lipoprotein Cholesterol; MMSE, Mini-Mental State Examination; MNA Mini Nutritional Assessment; PLTs, Platelets Count; POMA, Tinetti Performance-Oriented Mobility Assessment; PPT, Performance-Based Physical Test*;* PTH, Parathyroid hormone; RBCs, Red Blood Cells; SD, Standard Deviation;TG, triglycerides; WBCs, White blood cells.

### 3.1 Principal component analysis

We performed a PCA, and the first four PCs explained 73.5% of the total variance in the dataset. Significant Factor Loadings for the four PCs are reported in Table 2, while the relative contribution of each variable in the PCs is provided in Supplementary Table S1 **.**

- PC1, accounting for 34.9% of the total variance, was positively associated with comorbidity burden (CIRS Tot, CIRS ISC-13 and CIRS ISC-14) mood impairment (GDS), and negatively associated with cognitive performance (MMSE), nutritional status (MNA), functional independence (ADLs, IADLs), and educational attainment. This component appears to represent a *Frailty Axis*, reflecting the convergence of multimorbidity, depressive symptoms, cognitive and functional decline, and poor nutritional status, particulary among individuals with lower educational levels. Higher PC1 scores indicated increased frailty, whereas lower scores reflected greater clinical robustness.
- PC2, explaining 18.5% of the total variance, was positively associated with cognitive performance (MMSE), nutritional status (MNA), functional independence (ADLs, IADLs), educational level, as well ashigher comorbidity (CIRS Tot. CIRS ISC-13, CIRS ISC-14). This component may reflect a *Reserve Capacity Axis*, encompassing cognitive, nutritional, and functional resilience, possibly supported by higher education, even in the presence of comorbidities. Higher PC2 scores identified individuals with preserved global function and resources, despite an underlying burden of systemic comorbidities.
- PC3, accounting for 10.5% of the total variance, was primarily associated with mood impairment (GDS) and increased BMI, and negatively associated with age. This dimension may reflect a *Psychological–Metabolic Profile*, potentially identifying a subgroup of younger patients with depressive symptoms and higher body mass.
- PC4, explaining 9.9% of the total variance, was positively associated with education attainment and negatively with nutritional status (MNA) and BMI. This component might represent a subgroup of patients with higher educational attainment but lower nutritional reserves or body weight, potentially capturing nutritional vulnerability despite educational advantages.

**TABLE 2:** Significant factor loadings for the first four principal components.

Variable	PC1	PC2	PC3	PC4
Age (years)	NA	NA	−0.74	NA
Education (years)	−0.34	0.34	NA	0.60
MMSE	−0.31	0.62	NA	NA
GDS	0.46	NA	0.54	NA
ADLs	−0.69	0.40	NA	NA
IADLs	−0.62	0.53	NA	NA
MNA	−0.33	0.57	NA	−0.48
BMI	NA	NA	0.47	−0.64
CIRS	0.88	0.44	NA	NA
CIRS ISC-13	0.86	0.47	NA	NA
CIRS ISC-14	0.88	0.44	NA	NA

Only factor loadings with an absolute value ≥0.30 are reported in the table. Variables with lower loadings are indicated as “NA”.

Abbreviations: ADLs, Activities of Daily Living; BMI, Body Mass Index; CIRS, Cumulative Illness Rating Scale; CIRS ISC, Cumulative Illness Rating Scale Severity Index; GDS, Geriatric Depression Scale; IADLs, Instrumental Activities of Daily Living; MMSE, Mini-Mental State Examination; MNA, Mini Nutritional Assessment; NA, Not Applicable; PC, Principal Component.

**FIGURE 1:**
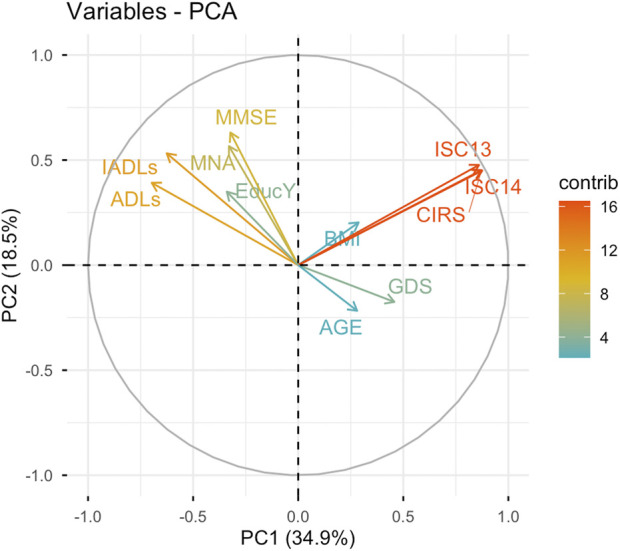
Mapping of variables into the First Two Principal Components. PC1 represented X axis; PC2 represented Y axis. The length of the arrows indicates the contribution of each variable along the axes: longer arrows signify greater contribution, while shorter arrows denote lesser contribution (as seen with BMI and AGE). PC1 accounts for 34.9% of the data variability. The variables positively contributing to this principal component include GDS, CIRS total score, CIRS ICS-13 and ICS-14. In contrast, the following variables have a negative contribution: Educational Level, MMSE, ADLs, IADLs and MNA. PC 2 explained the 18.5% of data variability and is positively associated with Educational Level, MMSE, ADLs, IADLs, MNA, CIRS, ISC-13 and ISC-14. Abbreviations: ADLs, Activities of Daily Living; BMI, Body Mass Index; CIRS, Cumulative Illness Rating Scale; CIRS ISC, Cumulative Illness Rating Scale Severity Index; GDS, Geriatric Depression Scale; IADLs, Instrumental Activities of Daily Living; MMSE, Mini-Mental State Examination; MNA,Mini Nutritional Assessment; PC, Principal Component; PCA, Principal Component Analysis.

The first two PCs, together accounting for 53.4% of the total variance, were used to represent the multivariate structure of the data in a biplot (Figure 1). This projection provided the basis for subsequent cluster analysis, allowing identification and visualization of groups of individuals with similar profiles.

### 3.2 Cluster analysis

A cluster analysis was subsequently performed. Both the dendrogram and silhouette method were examined to determine the optimal number of clusters (see Supplementary Figure S1). Although the average silhouette width suggested a marginally better compactness for a two-cluster solution (width = 0.20), a four-cluster configuration (width = 0.13) was selected based on its greater clinical interpretability, allowing for more meaningful differentiation of patient profiles when mapped onto the PCA-derived dimensions.

**FIGURE 2:**
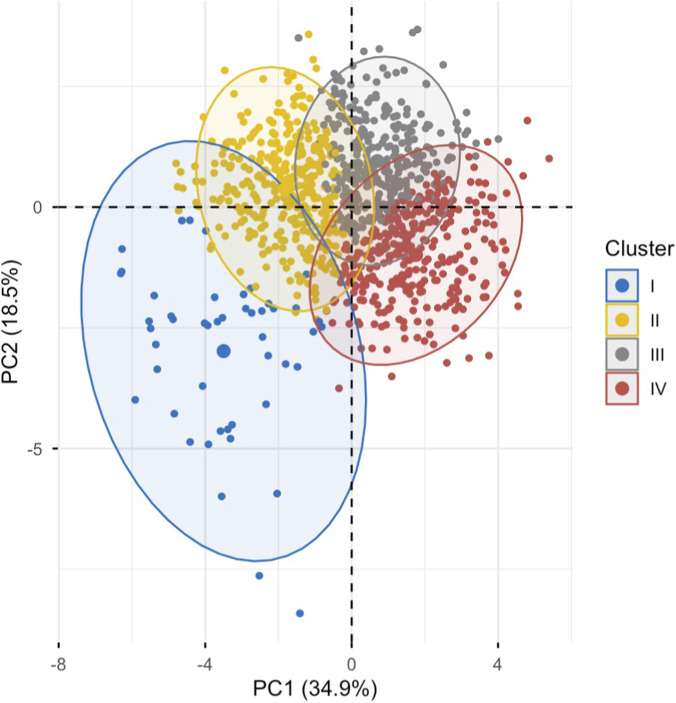
Cluster distribution of individuals according to principal components. Note: Cluster distribution of the sample based on principal component scores. The first two principal components (PC1 and PC2), accounting for 53.4% of the total variance, are used to project individuals into the PCA space. Each dot represents a patient, positioned in relation to each variable. There is a clear visual distinction between the clusters: Cluster 1 is represented by the blue area, Cluster 2 by the yellow area, Cluster 3 by the grey area, and Cluster 4 by the red area. Abbreviations: PC, Principal Component.

**FIGURE 3:**
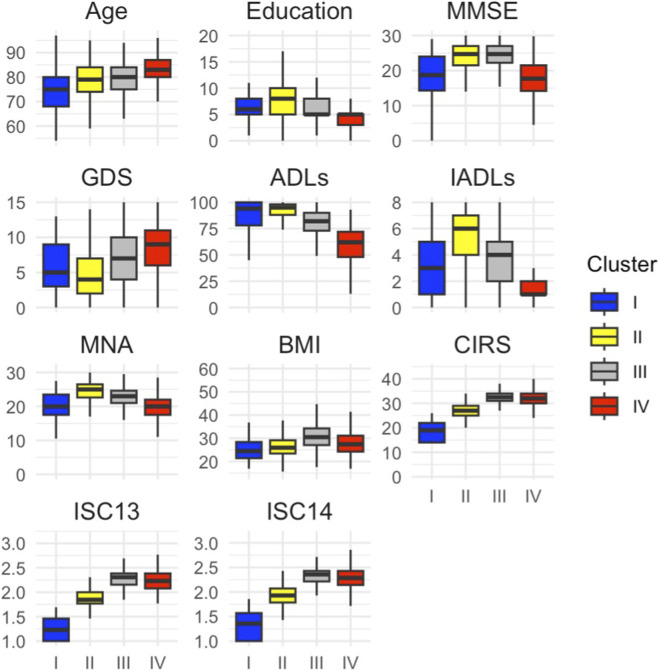
Boxplots of the variables included in the PCA, stratified by cluster. Note: Each box represents the distribution of a variable within the four clusters identified through hierarchical clustering on the PC. Variables include demographic, cognitive, functional, nutritional, and comorbidity measures. Age and Education: Cluster IV comprised the oldest participants, 82.92 (5.88) years, and had the lowest education levels, 4.82 (3.14) years. Conversely, Cluster I included the youngest participants, 73.90 (9.90) years, while Cluster 4 had higher education levels, 7.78 (4.22) years. Body Mass Index (BMI): The highest average BMI was observed in Cluster III, 31.06 (6.12), whereas Cluster I had the lowest, 25.79 (7.27). Cognitive Function (MMSE): Higher MMSE scores were observed in Clusters II and III, 24.32 (3.40) and 23.98 (4.13), respectively, suggesting relatively preserved cognitive function. In contrast, Clusters I and IV showed lower scores, 18.53 (6.72) and 17.64 (5.29), indicating greater cognitive impairment. Depressive Symptoms (GDS): Cluster IV showed the highest mean GDS score, 8.66 (3.61), indicating more depressive symptoms, while Cluster II had the lowest, 4.75 (3.19). Functional Status (ADLs and IADLs): Cluster II demonstrated the highest levels of independence, with ADLs and IADLs scores of 92.37 (8.74) and 5.48 (2.02), respectively. In contrast, Cluster IV had the lowest scores: ADLs 59.75 (15.58), IADLs 1.51 (1.16). Nutritional Status (MNA):Cluster II exhibited the highest mean MNA score, 24.36 (2.99), indicating better nutritional status, while Cluster I had the lowest, 17.54 (9.08). Comorbidity (CIRS, CIRS ISC-13, CIRS ISC-14): Clusters III and IV had higher CIRS scores, 32.62 (2.48) and 32.18 (3.22), respectively, indicating greater comorbidity burden, compared to Cluster I, 17.75 (4.70). Abbreviations: ADLs, Activities of Daily Living; BMI, Body Mass Index; CIRS, Cumulative Illness Rating Scale; CIRS ISC, Cumulative Illness Rating Scale Severity Index; GDS, Geriatric Depression Scale; IADLs, Instrumental Activities of Daily Living; MMSE, Mini-Mental State Examination; MNA, Mini Nutritional Assessment; PC, Principal Component; PCA, Principal Component Analysis.

The characteristics of the four clusters are illustrated in Figure 2, while Figure 3 presents boxplots of the PCA-derived variables across clusters. Table 3 provides a multidimensional characterization of the clusters, including significant *post-hoc* comparisons for additional clinical and laboratory variables not included in the original clustering model. A complete overview of all variables across clusters is presented in Supplementary Table S2.

**TABLE 3:** Post-hoc comparisons from multidimensional cluster characterization.

Variables	Cluster I (n = 49) Mean (SD) (z-score)	Cluster II (n = 346) Mean (SD) (z-score)	Cluster III (n = 344) Mean (SD) (z-score)	Cluster IV (n = 316) Mean (SD) (z-score)	p-value overall
CGA					
PPT	(n = 41) 13.00 (9.66) −0.16	(n = 344) 19.39 (5.61) +0.78	(n = 344) 13.70 (4.83) −0.08	(n = 313) 9.33 (4.16) −0.75	<0.0001^a^ 1.1.19
POMA 1.1.20	(n = 43) 16.98 (11.59) −0.01	(n = 344) 21.92 (5.28) +0.68	(n = 344) 16.60 (5.72) −0.06	(n = 314) 12.08 (5.92) −0.69	<0.0001^b^ 1.1.21
ESS	(n = 43) 10.88 (8.64) −1.04	(n = 343) 17.62 (2.48) +0.63	(n = 339) 15.47 (3.10) +0.05	(n = 309) 13.09 (2.34) −0.58	<0.0001^c^ 1.1.22
PRISMA 7	1.79 (1.34) −0.70	1.47 (1.13) −0.90	3.31 (1.11) +0.21	4.38 (1.14) +0.86	<0.0001^d^
Sub item CIRS					
Cardiovascular conditions	1.02 (0.14) −1.10	1.63 (0.80) −0.42	2.35 (0.84) +0.37	2.22 (0.89) +0.23	<0.0001^e^ 1.1.28
Hypertension	1.37 (0.73) −1.47	2.33 (0.91) −0.27	2.83 (0.52) +0.35	2.66 (0.71) +0.14	<0.0001^f^ 1.1.29
Vascular conditions	1.18 (0.56) −1.56	2.17 (0.79) −0.28	2.60 (0.64) +0.27	2.58 (0.67) +0.26	<0.0001^g^
Genitourinary conditions	1.22 (0.62) −1.39	1.98 (0.77) −0.42	2.48 (0.68) +0.23	2.63 (0.59) +0.43	<0.0001^h^
Musculoskeletal and dermatological disorders	1.63 (0.86) −1.68	2.56 (0.72) −0.50	3.11 (0.57) +0.19 1.1.30	3.43 (0.59) +0.60	<0.0001^i^
Psychiatric-behavioral disorders	2.02 (1.09) −1.34	2.61 (0.55) −0.30	2.83 (0.46) +0.08	3.05 (0.38) 0.46	<0.0001^j^ 1.1.31
Laboratory assessment					
Hb (g/dL)	(n = 31) 12.98 (1.50) +0.13	(n = 227) 13.05 (1.55) +0.17	(n = 236) 12.51 (1.86) −0.08	(n = 204) 12.39 (1.78) −0.13	0.0002^k^ 1.1.37
WBCs (x 10^3^/µL)	(n = 31) 6.88 (1.96) −0.01	(n = 228) 6.57 (1.96) −0.14	(n = 233) 6.97 (1.94) +0.02	(n = 204) 7.25 (2.19) +0.13	0.007^l^ 1.1.38
Serum creatinine (mg/dL)	(n = 27) 0.83 (0.17) −0.24	(n = 215) 0.90 (0.24) −0.18	(n = 227) 1.13 (0.68) +0.13	(n = 202) 1.10 (0.70) +0.09	<0.0001^m^ 1.1.39
BUN (mg/dL)	(n = 26) 32.01 (21.63) −0.10	(n = 148) 30.12 (13.12) −0.13	(n = 153) 37.74 (22.39) +0.07	(n = 143) 37.96 (31.81) +0.08	0.011^n^ 1.1.40
eGFR (mL/min/1.73 m^2^), CKD-EPI	(n = 27) 72.23 (17.18) +0.51	(n = 215) 69.55 (16.61) +0.25	(n = 227) 59.93 (20.85) −0.14	(n = 202) 58.34 (17.75) −0.20	<0.0001^o^ 1.1.41
Total cholesterol (mg/dL)	(n = 20) 195.65 (38.91) +0.06	(n = 158) 199.66 (45.78) +0.13	(n = 146) 183.31 (44.09) −0.12	(n = 128) 190.27 (42.13) −0.02	0.0135^p^ 1.1.42
HDL-C (mg/dL)	(n = 17) 57.59 (16.19) −0.01	(n = 151) 61.82 (15.06) +0.16	(n = 145) 54.25 (16.31) −0.15	(n = 122) 57.71 (15.54) −0.01	0.0007^q^ 1.1.43

Pairwise p-values are indicated below:

^a^ IvsII <0.0001; IvsIII = 0.84; IvsIV = 0.0001; IIvsIII <0.0001; IIvsIV <0.0001; IIIvsIV <0.0001.

^b^ IvsII <0.0001; IvsIII = 0.98; IvsIV <0.0001; IIvsIII <0.0001; IIvsIV <0.0001; IIIvsIV <0.0001.

^c^ IvsII <0.0001; IvsIII <0.0001; IvsIV = 0.0001; IIvsIII <0.0001; IIvsIV <0.0001; IIIvsIV <0.0001.

^d^ IvsII 0.376; IvsIII <0.0001; IvsIV = 0.0001; IIvsIII <0.0001; IIvsIV <0.0001; IIIvsIV <0.0001.

^e^ IvsII <0.0001; IvsIII <0.0001; IvsIV <0.0001; IIvsIII <0.0001; IIvsIV <0.0001; IIIvsIV = 0.197.

^f^ IvsII <0.0001; IvsIII <0.0001; IvsIV <0.0001; IIvsIII <0.0001; IIvsIV <0.0001; IIIvsIV = 0.016.

^g^ IvsII <0.0001; IvsIII <0.0001; IvsIV <0.0001; IIvsIII <0.0001; IIvsIV <0.0001; IIIvsIV = 0.99.

^h^ IvsII <0.0001; IvsIII <0.0001; IvsIV <0.0001; IIvsIII <0.0001; IIvsIV <0.0001; IIIvsIV = 0.02.

^i^ IvsII <0.0001; IvsIII <0.0001; IvsIV <0.0001; IIvsIII <0.0001; IIvsIV <0.0001; IIIvsIV <0.0001.

^j^ IvsII <0.0001; IvsIII <0.0001; IvsIV <0.0001; IIvsIII <0.0001; IIvsIV <0.0001; IIIvsIV <0.0001

^k^ IvsII = 0.99; IvsIII = 0.49; IvsIV = 0.28; IIvsIII = 0.004; IIvsIV = 0.0003; IIIvsIV = 0.86.

^l^ IvsII = 0.85; IvsIII = 0.99; IvsIV = 0.79; IIvsIII = 0.14; IIvsIV = 0.003; IIIvsIV = 0.47.

^m^ IvsII =0.95; IvsIII = 0.06; IvsIV =0.11; IIvsIII = 0.0002; IIvsIV = 0.002; IIIvsIV = 0.95.

^n^ IvsII =0.98; IvsIII = 0.65; IvsIV =0.63; IIvsIII = 0.026; IIvsIV =0.023; IIIvsIV =0.99.

^o^ IvsII =0.10; IvsIII = 0.0001; IvsIV = 0.0001; IIvsIII = 0.0001; IIvsIV <0.0001; IIIvsIV = 0.81.

^p^ IvsII =0.98; IvsIII = 0.64; IvsIV = 0.56; IIvsIII = 0.0069; IIvsIV = 0.27; IIIvsIV = 0.96.

^q^ IvsII =0.72; IvsIII =0.84; IvsIV =0.27; IIvsIII = 0.0002; IIvsIV = 0.13; IIIvsIV = 0.99.

Data are presented as mean (SD) and z-scores. Post-hoc comparisons between clusters were performed for selected variables using ANOVA test, with Bonferroni correction for multiple testing; p-values <0.05 were considered statistically significant.

Abbreviations: BUN, Blood Urea Nitrogen; CGA, Comprehensive Geriatric Assessment; CIRS, Cumulative Illness Rating Scale; eGFR, Estimated Glomerular Filtration Rate; CKD, Chronic Kidney Disease Epidemiology Collaboration; ESS, Exton-Smith Scale; Hb, Haemoglobin; HDL-C, High-density lipoprotein Cholesterol; n, number of participants; POMA, Tinetti Performance-Oriented Mobility Assessment; PPT, Performance-Based Physical Test; SD, Standard Deviation; WBs, White blood cells.

#### 3.2.1 Cluster I- Vulnerable Low-Complexity Phenotype (PC1−/PC2−; lower left quadrant)

This cluster comprises younger individuals (z = −0.85), with low comorbidity burden (CIRS Tot. z = −2.58; CIRS ISC-13/14 z ≈ −2.5) and adequate educational attainment (z = +0.18) yet marked deficits across multiple domains.

- Cognitive and Mood Profile: MMSE scores below average (z = −0.63), while absent depressive symptoms (GDS z = −0.26).
- Functional Status: moderate dependence in both ADLs (z = +0.40) and IADLs (z = −0.06) scales; Moderate dependence at PPT (z = −0.16), significantly better than Cluster IV but worse than Cluster III (all p < 0.0001); Gait performance (POMA z = −0.01) with a high fall risk, worse than Cluster III and IV (all p < 0.0001).
- Nutritional Domain and Skin Risk: clear malnutrition (MNA z = −1.06) with relatively low BMI (z = −0.43), and the highest risk of pressure ulcers among all clusters (ESS z = −1.04; p < 0.0001).
- Laboratory Profile: Mean serum albumin levels within normal limits, but significantly higher than in Cluster III (p = 0.0066) and IV (p = 0.0022).

Although patients in this cluster exhibit low overall clinical complexity and no overt depressive symptoms, they display a combination of cognitive impairment, functional vulnerability, malnutrition, and high-pressure sore risk, defining a *vulnerable low-complexity phenotype*.

#### 3.2.2 Cluster II - Resilient High-Reserve Phenotype (PC1-/PC2+; upper left quadrant)

This cluster includes older adults with high educational attainment (z = +0.36), preserved cognitive, functional, and nutritional capacities, low depressive symptoms, low comorbidity burden and the lowest frailty risk.

- Cognitive and Mood Profile: higher MMSE scores (z = +0.38) and the lowest GDS scores (z = −0.52), indicating intact cognition and preserved mood.
- Functional Status: elevated score in ADLs (z = +0.74) and IADLs (z = +0.78) scale, combined with superior physical performance on the PPT (z = +0.78) and POMA (z = +0.68), reflecting minimal functional dependence, low disability, and reduced fall risk (all p < 0.0001 vs. other clusters).
- Nutritional Domain and Skin Risk: optimal nutritional profile with the highest MNA scores (z = +0.53), and lowest risk of pressure sores (ESS z = +0.63) across all clusters (all p < 0.0001), with only approximately 1.5% of individuals scoring below the ESS threshold (<13) (χ ^2^ = 206.547; p < 0.0001).
- Frailty profile:low frailty burden (z = −0.90) compared to Cluster III and IV (p < 0.0001).
- Comorbidity Burden: low overall comorbidity (CIRS Tot. z = −0.65), with relatively low prevalence of major organ-specific conditions compared to Clusters III and IV (p < 0.0001).
- Laboratory Profile: significantly higher haemoglobin levels (z = +0.17, p = 0.0003) and lower WBCs (z = −0.14, p = 0.003), serum creatinine (z = −0.18, p = 0.002) and BUN (z = −0.13, p = 0.023) compared to Cluster IV; higher total cholesterol (z = +0.13, p = 0.0069) and HDL-C (z = +0.16, p = 0.0002) compared to Cluster III.

Therefore, Cluster II represents a *resilient high-reserve phenotype*, characterized by high levels of cognitive, physical, and nutritional function and low comorbidity burden.

#### 3.2.3 Cluster III- Resilient Frailty Phenotype (PC1+/PC2+; upper right quadrant)

This cluster is defined by elevated comorbidity, mild depressive symptoms, functional and nutritional vulnerability, including increased risk of pressure sores, and metabolic imbalance, despite relatively preserved cognitive reserve.

- Comorbidity: elevated CIRS Tot. (z = +0.58), CIRS ISC-13 (+0.61), and CIRS ISC-14 (+0.59); higher prevalence of cardiovascular (z = +0.37), hypertensive (z = +0.35), respiratory (z = +0.31), upper gastrointestinal (z = +0.36), and endocrine-metabolic diseases (z = +0.33) (all p < 0.001).
- Cognition and mood: MMSE above average (z = +0.44); GDS mildly elevated (z = +0.10).
- Functional domain:
o ADLs/IADLs scores above the sample mean (z = +0.13/+0.04) but still indicative of moderate dependence.
o Moderate disability at PPT (z = −0.08), significantly better than Cluster IV (p < 0.0001) but worse than Cluster II (p < 0.0001).
o Gait performance (POMA), indicating fall risk (z = −0.06), better than Cluster IV (p < 0.0001), worse than Cluster II (p < 0.0001).
- Nutrition: risk of malnutrition (MNA z = +0.17) accompanied by elevated BMI (z = +0.46), suggesting obesity.
- Pressure ulcer risk: mild (ESS z = +0.05), lower than Cluster II and IV (p < 0.0001), higher than Cluster I (p < 0.0001).
- Laboratory profile: higher creatinine (z = +0.13), and BUN (z = +0.07) levels than Cluster II (both p < 0.05); lower total cholesterol (z = −0.12, p = 0.0069) and HDL-C (z = −0.15, p = 0.0002) vs. cluster II, although mean values remained within normal ranges.

Despite increased frailty indicators (PC1+), their higher PC2 scores a *resilient frailty phenotype*, characterized by a substantial burden of chronic disease alongside compensatory cognitive reserve.

#### 3.2.4 Cluster IV- Globally Frail Phenotype (PC1+/PC2–; lower right quadrant)

Cluster IV represents the oldest subgroup (z = +0.43) and is characterized by the most compromised profile across nearly all domains.

• Cognitive and Mood domains: the lowest cognitive performance across clusters (MMSE z = −0.80), mild-to-moderate depressive symptoms (GDS z = +0.50) significantly worse than all other clusters (p < 0.0001).
• Functional Profile: marked functional dependence (ADLs z = −1.01; IADLs z = −0.89), impaired Physical performance, reflected by low PPT scores (z = −0.75) with 99% scoring belowthe PPT threshold (<21; χ ^2^ = 253.303; p < 0.0001), poor gait performance (POMA z = −0.69),with 97.5% below the <25 cut-off (χ ^2^ = 182.266; p < 0.0001).
• Nutritional Domain and Skin Risk: malnutrition risk (z = −0.60), elevated pressure ulcer risk (ESS z = −0.58), with 39.2% of participants below the ESS cutoff (<13) (χ^2^ = 206.547; p < 0.0001).
• Comorbidity burden: high comorbidity scores (CIRS Tot. z = +0.49), particularly for genitourinary (z = +0.43), musculoskeletal (z = +0.60) and neuropsychiatric (z = +0.46) conditions (all p ≤ 0.02 vs. all clusters).
• Frailty burden: marked frailty risk, evidenced by the highest PRISMA-7 scores across clusters (mean 4.38, z = +0.86; p < 0.0001) and by 93% of individuals exceeding the frailty threshold (χ^2^ = 457.238; p < 0.0001).
• Laboratory Profile: lower haemoglobin (z = −0.13, p = 0.0003)compared to Cluster II, although all values within normal reference ranges. Additionally, approximately 55% of individuals exhibited impaired renal function (eGFR <60 mL/min/1.73 m^2^; χ^2^ = 35.17; p < 0.0001),

Overall, this cluster reflects a *globally frail phenotype* marked by advanced age, cognitive, mood and functional impairment, poor nutritional status and a high burden of chronic disease.

## 4 Discussion

The present study provides a comprehensive overview of the clinical heterogeneity observed in a cohort of 1055 older outpatients. The PCA-based multidimensional space allowed us to capture the interplay between *Frailty Axis* (PC1) and *Reserve Capacity Axis* (PC2), which served as the basis for identifying four distinct phenotypic clusters through Cluster Analysis.

One of the most salient findings is the pivotal role played by *Reserve Capacity*, which encompasses cognitive, functional and nutritional reserve, potentially reinforced by higher education. Our results showed that individuals with greater reserve capacity and higher levels of education were significantly more likely to belong to Cluster II (“*Resilient High-Reserve Phenotype*”). The findings are consistent with a growing body of literature emphasizing the contribution of education to reserve building and its protective role against age-related cognitive and functional decline (Meng and D'Arcy, 2012; Scarmeas and Stern, 2003; Stern, 2009; Tucker and Stern, 2011; Valenzuela and Sachdev, 2006a). Collectively, these observations underscore the critical importance of promoting educational opportunities across the lifespan as a strategy to enhance reserve and support healthy aging.

However, the profile of Cluster I (“*Vulnerable Low-Complexity Phenotype*”) underscores the multidimensional nature of reserve capacity. Despite moderate educational attainment, individuals in this cluster presented low reserve, as evidenced by early signs of cognitive and functional vulnerability. This finding suggests that reserve capacity is not solely determined by education but is shaped by a constellation of interacting factors. Indeed, previous research has highlighted the role of additional socioeconomic determinants, such as engagement in leisure activities, occupational complexity, and income, in the development and maintenance of reserve (Chen et al., 2022; Foubert-Samier et al., 2012; Lane et al., 2017).

In our PCA, Reserve Capacity was also conceptualized in terms of nutritional reserve, as evidenced by the positive loading of the MNA on PC2, as well as by inter-cluster differences. Notably, Clusters I and IV, both characterized by low reserve, showed the poorest performances not only on the MNA but also on the ESS scale. Specifically, Clusters I exhibited clear signs of malnutrition, the lowest BMI values and the highest risk for pressure ulcers. These findings are consistent with previous research highlighting the central role of the nutritional statusin shaping physical reserve (Bernstein et al., 2012; Buys et al., 2014; Porter Starr et al., 2015; Yukawa et al., 2014).

Regarding the Frailty Axis, our findings indicate that frail patients, included in Cluster III (the “Resilient Frailty Phenotype”, characterized byhigh reserve capacity) and Cluster IV (the “Globally Frail Phenotype”, characterized by low reserve capacity) exhibited a significantly higher comorbidity burden, both in terms of overall severity and across multiple specific CIRS subscales. These observations are well-documented in the literature, where frailty is strongly associated with the accumulation of chronic conditions affecting multiple physiological systems, including cardiovascular, respiratory, renal, musculoskeletal, and neuropsychiatric domains (Gobbens et al., 2024; Ogaz-González et al., 2024). Furthermore, frail individuals showed lower glomerular filtration rates, corresponding to Kidney DiseaseImproving Global Outcomes (KDIGO) 2024 Stage IIIA chronic kidney disease (45–59 mL/min/1.73 m^2^), whereas individuals in Clusters I and II, characterized by lower levels of frailty, were more frequently classified within Stage II (60–89 mL/min/1.73 m^2^). These findings align with previous research suggesting that systemic inflammation serves as a shared pathophysiological mechanism linking frailty and chronic kidney disease. Elevated levels of pro-inflammatory cytokines, such as interleukin-6 and tumor necrosis factor-alpha, have been consistently implicated in the pathogenesis and progression of chronic kidney disease, contributing to renal inflammation, fibrosis, and accelerated decline in glomerular filtration rate (Hubbard and Woodhouse, 2010; Jeffery et al., 2013; Shlipak et al., 2003).

Additionally, frail individuals exhibited significantly lower levels of Total cholesterol and HDL-C, a finding that may be attributed to chronic low-grade inflammation and aligns with the so-called “lipid paradox”, whereby lower cholesterol levels paradoxically associate with increased morbidity and mortality in frail populations (Turusheva et al., 2020). This phenomenon has been attributed to the effects of systemic inflammation and underlying chronic illness, which can reduce circulatingcholesterol levels independently of cardiovascular risk (Ravnskov et al., 2016; Turusheva et al., 2020). In contrast, no significant association in LDL-C levels were observed across clusters. The absence of significant variation in TG levels may partly explain this lack of difference. Since LDL-C was estimated using the Friedewald formula, which includes triglycerides in its computation, the limited variability in TG values likely limited the capacity to detect between-cluster differences in the estimated LDL-C levels.

Within frail clusters, those with low reserve (Cluster I) showed a lower prevalence of hypertension, respiratory, and upper gastrointestinal disorders compared to frail individuals with high reserve. This apparent paradox may reflect underdiagnosis related to reduced healthcare utilization (Zhao et al., 2022), as well as atypical or attenuated clinical presentations commonly observed in individuals with impaired cognitive and functional reserves (Jarrett et al., 1995; Limpawattana et al., 2016). Rather than indicating a truly lower disease burden, the low comorbidity scores may reflect a form of latent vulnerability that becomes evident only when adopting a multidimensional approach.

Conversely, frail individuals with low reserve capacity (Cluster IV) exhibited a greater burden of genitourinary, musculoskeletal, and neuropsychiatric conditions, chronic issues typically encompassed within the construct of geriatric syndromes, which require intact functional and cognitive reserves for adequate compensation. These findings are consistent with prior literature, reinforcing the concept that geriatric syndromes tend to cluster in the most vulnerable older adults (Anzaldi et al., 2017). Notably, individuals in this cluster were the oldest in the sample. This observation aligns with a recent systematic review and meta-analysis demonstrating a robust association between advancing age and frailty, supporting the concept of a “frailty cycle” (Clegg and Young, 2011), a progressive process driven by the interplay of multiple factors, including non-modifiable ones such as biological aging.

In this context, our findings highlight the utility of CGA as a robust tool for the multidimensional profiling of older adults. The present study illustrates the potential of CGA, when combined with multivariate statistical techniques, to delineate clinically meaningful profiles. Several studies have supported the use of CGA in guiding clinical decision-making and optimizing care pathways in older populations (Brigg et al., 2022; Disalvo et al., 2025; Ellis et al., 2011; Pilotto et al., 2010; Rubenstein et al., 1991).

Although PCA and cluster analysis were conducted *post-hoc* in the present study, their translation into routine clinical practice is feasible. The CGA dimensions identified as discriminative could be incorporated into a decision-tree–like guideline to support manual profiling during bedside assessment. Such an approach would allow clinicians not only to identify heterogeneous frailty phenotypes but also to design tailored interventions. In this context, PC2 emerges as a promising axis for strategic intervention, encompassing cognitive training and stimulation (Valenzuela and Sachdev, 2006b; Wenisch et al., 2007; Wilson et al., 2002), physical rehabilitation (American College of Sports Medicine et al., 2009), nutritional optimization (Volkert et al., 2019), and lifelong learning programs (Gangemi et al., 2023; Narushima et al., 2018), which may act synergistically to increase reserve capacity. By strengthening these domains, it may be possible to shift individuals toward more favorable phenotypic zones along the PC2 axis, potentially mitigating the risk of frailty progression.

This study has several limitations. First, its cross-sectional design precludes any inference on causality or temporal evolution between the observed clusters and clinical outcomes. While CGA-based stratification may support the development of multidomain preventive and rehabilitative interventions tailored to older patient profiles, interventions aimed at enhancing reserve capacity require longitudinal validation. Moreover, although our findings suggest a role for educational attainment in reserve building, further research is needed to clarify the broader set of biological, social, and lifestyle factors contributing to reserve capacity. Second, selection bias cannot be excluded, as the study population comprised older adults attending outpatient geriatric services, potentially underrepresenting individuals with more severe impairment or those unable to access ambulatory care. Although the average silhouette width marginally favored a two-cluster solution (0.20), a four-cluster model (0.13) was selected based on its superior clinical interpretability and its ability to reflect the heterogeneity of the population. Finally, the relatively small size of Cluster It represents a potential limitation, as imbalanced cluster sizes may introduce bias and affect the stability and generalizability of cluster-based findings.

## 5 Conclusion

This study provides an innovative approach to patient profiling in geriatrics through PCA-informed cluster analysis of CGA data. Our findings highlight CGA as a valuable tool for stratifying older adults, with indicators such as educational attainment emerging as key contributors to clinical reserve. By identifying distinct phenotypic patterns based on the interplay between frailty and reserve, this study contributes to a deeper understanding of the clinical heterogeneity in older adults and underscores the relevance of reserve capacity in geriatric profiling.

## Data Availability

The raw data supporting the conclusions of this article will be made available by the authors, without undue reservation.

## Glossary

ADLs: Activities of Daily Living
ALB: Albumin
BUN: Blood Urea Nitrogen
CGA: Comprehensive Geriatric Assessment
CIRS Tot.: Cumulative Illness Rating Scale Total Score
CIRS ISC: CIRS Severity Index
CIRS ISC-13: CIRS Severity Index, average of the first 13 items
CIRS ISC-14: CIRS Severity Index, average of all items
CI: Confidence Interval
CKD-EPI: Chronic Kidney Disease Epidemiology Collaboration
eGFR: Estimated Glomerular Filtration
ESS: Exton-Smith Scale
FBG: Fibrinogen
GDS: Geriatric Depression Scale
Hb: Haemoglobin
HDL-C: High-Density LipoproteinCholesterol
IADLs: Instrumental Activities of Daily Living
ISTAT: Italian National Institute of Statistics
KDIGO: Kidney Disease Improving Global Outcomes
LDL-C: Low-Density Lipoprotein Cholesterol
MNA: Mini Nutritional Assessment
MMSE: Mini-Mental State Examination
PCA: Principal Component Analysis
PCs: Principal Components
PLT: Platelet Count
POMA: Tinetti Performance-Oriented Mobility Assessment
PPT: Performance-Based Physical Test
PTH: Parathyroid Hormone
RBCs: Red Blood Cell Count
SD: Standard Deviation
TG: Triglycerides
TP: Total Protein
WBCs: White Blood Cell Count
WHO: World Health Organization
