# Supplementary material for: Data-driven frailty and reserve phenotypes in older outpatients: a cluster analysis of Comprehensive Geriatric Assessment
Source: Front Aging. 2026 Jan 12;6:1678407. doi: 10.3389/fragi.2025.1678407 (PMC12833285; doi:10.3389/fragi.2025.1678407)
Supplement: Supplementary file 1 [file Table1.docx]

**Supplementary Materials**

**Table S1:**

Contributions (%) of each variable to the first four principal components (PCs)

| \| Variable \| PC1 (%) \| PC2 (%) \| PC3 (%) \| PC4 (%) \| \| --- \| --- \| --- \| --- \| --- \| \| *Age (years)* \| 2.08 \| 2.48 \| 47.74 \| 0.74 \| \| *Education (years)* \| 2.98 \| 5.61 \| 1.12 \| 33.45 \| \| *MMSE* \| 2.43 \| 19.04 \| 0.36 \| 3.54 \| \| *GDS* \| 5.40 \| 1.58 \| 25.01 \| 1.52 \| \| *ADLs* \| 12.52 \| 8.09 \| 0.96 \| 0.08 \| \| *IADLs* \| 10.10 \| 14.26 \| 0.26 \| 0.08 \| \| *MNA* \| 2.77 \| 16.16 \| 3.88 \| 21.01 \| \| *BMI* \| 2.12 \| 2.22 \| 18.88 \| 37.64 \| \| *CIRS* \| 19.99 \| 9.86 \| 0.60 \| 0.58 \| \| *CIRS ISC-13* \| 19.46 \| 10.94 \| 0.67 \| 0.70 \| \| *CIRS ISC-14* \| 20.15 \| 9.77 \| 0.53 \| 0.65 \|   Abbreviations: **ADLs**: Activities of Daily Living; **BMI**: Body Mass Index; **CIRS**: Cumulative Illness Rating Scale; **CIRS ISC**, Cumulative Illness Rating Scale Severity Index; **GDS**: Geriatric Depression Scale; **IADLs**: Instrumental Activities of Daily Living; **MMSE**: Mini-Mental State Examination; **MNA** Mini Nutritional Assessment; **PC:** Principal Component |
| --- | --- | --- | --- | --- | --- | --- | --- | --- | --- | --- | --- | --- | --- | --- | --- | --- | --- | --- | --- | --- | --- | --- | --- | --- | --- | --- | --- | --- | --- | --- | --- | --- | --- | --- | --- | --- | --- | --- | --- | --- | --- | --- | --- | --- | --- | --- | --- | --- | --- | --- | --- | --- | --- | --- | --- | --- | --- | --- | --- | --- |

| **Figure S1:**  Visual (**A**) and silhouette method (**B**) to determine the optimal number of clusters (k) | |  |
| --- | --- | --- |
|  | |  |
| **(A)**  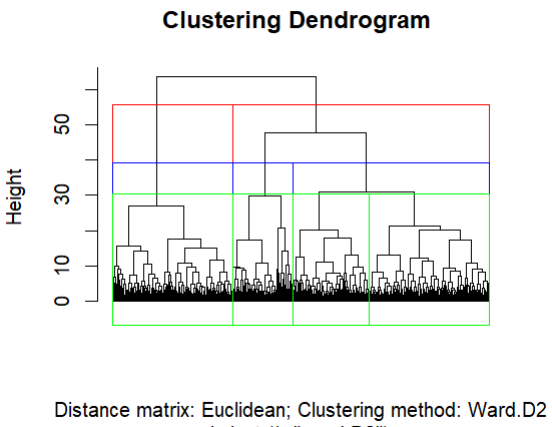 | | **(B)**  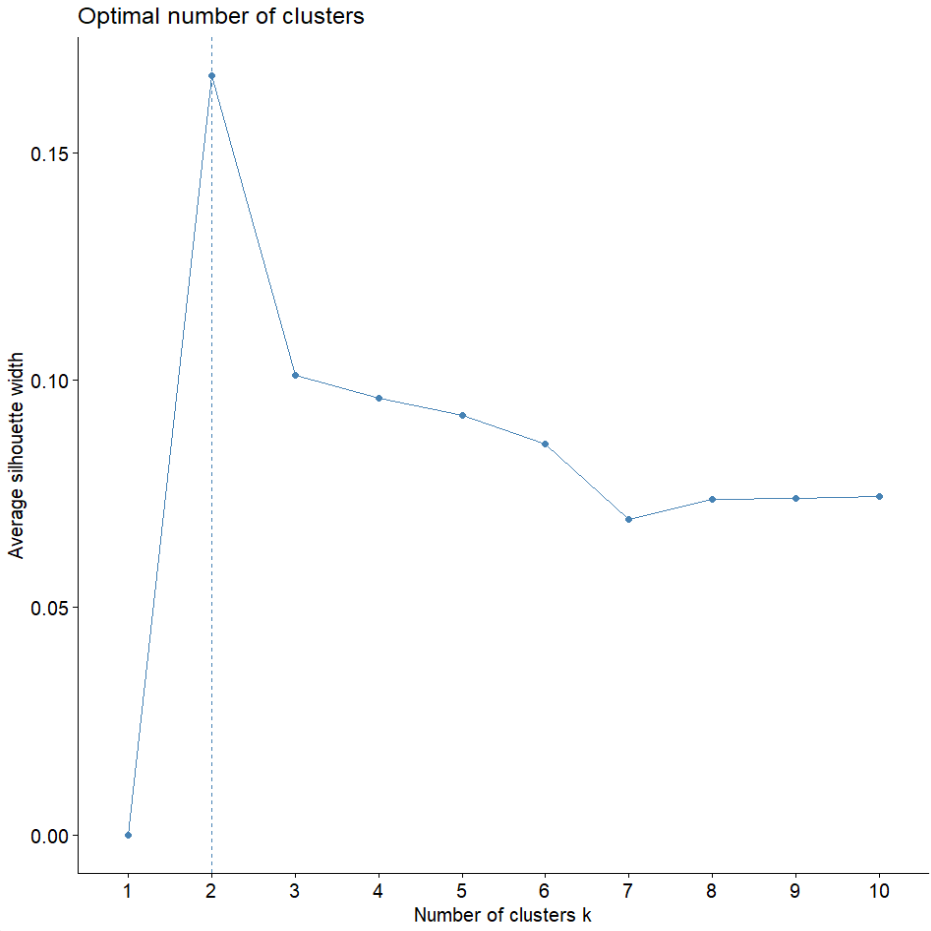 |
| **Note**: The dendrogram on the left shows different cut levels (k = 2 in red, k = 3 in green, and k = 4 in blue). The plot on the right displays the average silhouette width for k = 1 to 10 clusters.  K = number of clusters | |  |

**Table S2** Multidimensional characterization of clusters with post-hoc comparisons

| Variables | **Cluster I**  **(n = 49)**  Mean (SD)  (z-score) | **Cluster II**  **(n = 346)**  Mean (SD)  (z-score) | **Cluster III**  **(n = 344)**  Mean (SD)  (z-score) | **Cluster IV**  **(n=316)**  Mean (SD)  (z-score) | p-value overall |
| --- | --- | --- | --- | --- | --- |
| **Demographic Data** |  |  |  |  |  |
| *Age, | 73.90 (9.90)  **-0.85** | 78.54 (7.02) -0.19 | 79.31 (6.35) -0.08 | 82.92 (5.88) **+0.43** |  |
| *Education (years) | 7.10 (4.16)  +0.18 | 7.78 (4.22)  **+0.36** | 6.34 (3.43)  -0.01 | 4.83 (3.14)  **-0.41** |  |
| *BMI | 25.79 (7.27)  **-0.43** | 26.24 (4.31)  -0.36 | 31.07 (6.12)  **+0.46** | 28.11 (5.87)  -0.04 |  |
| **CGA** |  |  |  |  |  |
| *MMSE | 18.53 (6.72)  -0.63 | 23.98 (4.13)  +0.38 | 24.32 (3.40)  **+0.44** | 17.64 (5.29)  **-0.80** |  |
| *GDS | 5.78 (3.88)  -0.26 | 4.75 (3.19)  **-0.52** | 7.15 (3.58)  +0.10 | 8.66 (3.61)  **+0.50** |  |
| *ADLs | 86.12 (17.32)  +0.40 | 92.37 (8.74)  **+0.74** | 81.13 (11.44)  +0.13 | 59.75 (15.58)  **-1.01** |  |
| *IADLs | 3.49 (2.51)  -0.06 | 5.48 (2.02)  **+0.78** | 3.73 (1.83)  +0.04 | 1.51(1.16)  **-0.89** |  |
| PPT | (n = 41)  13.00 (9.66)  -0.16 | (n = 344)  19.39 (5.61)  **+0.78** | (n = 344)  13.70 (4.83)  -0.08 | (n = 313)  9.33 (4.16)  **-0.75** | **<0.0001** |
| POMA | (n = 43)  16.98 (11.59)  -0.01 | (n = 344)  21.92 (5.28)  **+0.68** | (n = 344)  16.60 (5.72)  -0.06 | (n = 314)  12.08 (5.92)  **-0.69** | **<0.0001** |
| *MNA | 17.54 (9.08)  **-1.06** | 24.36 (2.99)  **+0.53** | 22.81 (2.94)  +0.17 | 19.49 (3.70)  -0.60 |  |
| ESS | (n = 43)  10.88 (8.64)  **-1.04** | (n = 343)  17.62 (2.48)  **+0.63** | (n = 339)  15.47 (3.10)  +0.05 | (n = 309)  13.09 (2.34)  -0.58 | **<0.0001** |
| *CIRS | 17.75 (4.70)  **-2.58** | 26.81 (2.55)  -0.65 | 32.62 (2.48)  +0.58 | 32.18 (3.22)  +0.49 |  |
| *ISC-13 | 1.23 (0.24)  -2.52 | 1.86 (0.19)  -0.66 | 2.29 (0.19)  +0.61 | 2.24 (0.25)  +0.45 |  |
| *ISC-14 | 1.29 (0.29)  -2.55 | 1.92 (0.18)  -0.66 | 2.33 (0.18)  +0.59 | 2.30 (0.30)  +0.48 |  |
| PRISMA 7 | 1.79 (1.34)  -0.70 | 1.47 (1.13)  **-0.90** | 3.31 (1.11)  +0.21 | 4.38 (1.14)  **+0.86** | **<0.0001** |
| **Sub item CIRS:** |  |  |  |  |  |
| Cardiovascular conditions | 1.02 (0.14)  **-1.10** | 1.63 (0.80)  -0.42 | 2.35 (0.84)  **+0.37** | 2.22 (0.89)  +0.23 | **<0.0001** |
| Hypertension | 1.37 (0.73)  **-1.47** | 2.33 (0.91)  -0.27 | 2.83 (0.52)  **+0.35** | 2.66 (0.71)  +0.14 | **<0.0001** |
| Vascular conditions | 1.18 (0.56)  **-1.56** | 2.17 (0.79)  -0.28 | 2.60 (0.64)  **+0.27** | 2.58 (0.67)  +0.26 | **<0.0001** |
| Respiratory conditions | 1.10 (0.42)  **-0.56** | 1.33 (0.61)  -0.26 | 1.77 (0.86)  **+0.31** | 1.55 (0.76)  +0.03 | **<0.0001**^1^ |
| Sensory organ disorders (ENT & eye) | 1.59 (0.96)  **-1.45** | 2.45 (0.64)  -0.20 | 2.70 (0.57)  +0.17 | 2.77 (0.64)  **+0.26** | **<0.0001**^2^ |
| Upper gastrointestinal conditions | 1.06 (0.32)  **-1.04** | 1.62 (0.74)  -0.31 | 2.13 (0.72)  **+0.36** | 1.94 (0.73)  +0.10 | **<0.0001**^3^ |
| Lower gastrointestinal conditions | 1.10 (0.47)  **-1.06** | 1.66 (0.68)  -0.33 | 2.08 (0.73)  +0.23 | 2.11 (0.75)  **+0.27** | **<0.0001**^4^ |
| Hepatic conditions | 0.98 (0.14)  **-0.57** | 1.21 (0.47)  -0.14 | 1.40 (0.61)  +0.20 | 1.30 (0.57)  +0.02 | **<0.0001**^5^ |
| Renal conditions | 1.04 (0.35)  **-0.66** | 1.30 (0.55)  -0.27 | 1.64 (0.71)  **+0.24** | 1.60 (0.70)  +0.13 | **<0.0001**^6^ |
| Genitourinary conditions | 1.22 (0.62)  **-1.39** | 1.98 (0.77)  -0.42 | 2.48 (0.68)  +0.23 | 2.63 (0.59)  **+0.43** | **<0.0001** |
| Musculoskeletal and dermatological disorders | 1.63 (0.86)  **-1.68** | 2.56 (0.72)  -0.50 | 3.11 (0.57)  +0.19 | 3.43 (0.59)  **+0.60** | **<0.0001** |
| Neurological conditions | 1.10 (0.42)  **-0.74** | 1.52 (0.71)  -0.22 | 1.88 (0.86)  **+0.22** | 1.80 (0.83)  +0.12 | **<0.0001**^7^ |
| Endocrine-metabolic conditions | 1.33 (0.75)  **-1.53** | 2.44 (0.82)  -0.13 | 2.80 (0.57)  **+0.33** | 2.56 (0.78)  +0.02 | **<0.0001**^8^ |
| Psychiatric-behavioral disorders | 2.02 (1.09)  -**1.34** | 2.61 (0.55)  -0.30 | 2.83 (0.46)  +0.08 | 3.05 (0.38)  **0.46** | **<0.0001** |
| **Laboratory Assessment** |  |  |  |  |  |
| RBCs (x 10^3^/µL) | (n = 31)  4.59 (0.69)  -0.08 | (n = 227)  4.72 (0.71)  +0.06 | (n = 234)  4.62 (0.69)  -0.05 | (n = 202)  4.67 (0.76)  +0.00 | 0.45^9^ |
| Hb (g/dL) | **(**n = 31)  12.98 (1.50)  +0.13 | (n = 227)  13.05 (1.55)  **+0.17** | **(**n = 236)  12.51 (1.86)  -0.08 | **(**n = 204)  12.39 (1.78)  **-0.13** | **0.0002** |
| WBCs (x 10^3^/µL) | (n = 31)  6.88 (1.96)  -0.01 | (n = 228)  6.57 (1.96)  **-0.14** | (n = 233)  6.97 (1.94)  +0.02 | (n = 204)  7.25 (2.19)  **+0.13** | **0.007** |
| PLTs (x 10^3^/µL) | **(**n = 29)  219.86 (77.24)  -0.16 | (n = 226)  243.92 (76.21)  +0.08 | **(**n = 238)  234.06 (73.24)  -0.03 | (n = 202)  233.21 (74.98)  -0.03 | 0.23^10^ |
| Glucose (mg/dL) | (n = 25)  103.80 (23.17)  -0.11 | (n = 189)  109.12 (35.39)  +0.00 | (n = 176)  110.02 (32.10)  +0.02 | (n = 174)  108.88 (36.80)  -0.00 | 0.86^11^ |
| Serum Creatinine (mg/dL) | (n = 27)  0.83 (0.17)  **-0.24** | (n = 215)  0.90 (0.24)  -0.18 | (n = 227)  1.13 (0.68)  **+0.13** | **(**n = 202)  1.10 (0.70)  +0.09 | **<0.0001** |
| BUN (mg/dL) | (n = 26)  32.01 (21.63)  -0.10 | (n = 148)  30.12 (13.12)  **-0.13** | (n = 153)  37.74 (22.39)  +0.07 | (n = 143)  37.96 (31.81)  **+0.08** | **0.011** |
| eGFR (mL/min/1.73 m²), CKD-EPI | (n = 27)  72.23 (17.18) **+0.51** | (n = 215)  69.55 (16.61)  +0.25 | (n = 227)  59.93 (20.85)  -0.14 | (n = 202)  58.34 (17.75)  **-0.20** | **<0.0001** |
| Total Protein (g/dL) | (n = 13)  7.01 (0.41)  +0.01 | (n = 103)  7.08 (0.56)  +0.08 | (n = 129)  7.01 (0.65)  +0.01 | (n = 128)  6.89 (0.57)  -0.10 | 0.13^12^ |
| Albumine (g/dL) | (n = 14)  3.87 (0.49)  -0.02 | (n = 127)  4.02 (0.38)  **+0.21** | (n = 131)  3.85 (0.34)  -0.05 | (n = 110)  3.77 (0.41)  **-0.17** | **<0.0001**^13^ |
| Total Cholesterol (mg/dL) | (n = 20)  195.65 (38.91)  +0.06 | (n = 158)  199.66 (45.78)  **+0.13** | (n = 146)  183.31 (44.09)  **-0.12** | (n = 128)  190.27 (42.13)  -0.02 | **0.0135** |
| HDL-C (mg/dL) | (n = 17)  57.59 (16.19)  -0.01 | (n = 151)  61.82 (15.06)  **+0.16** | (n = 145)  54.25 (16.31)  **-0.15** | (n = 122)  57.71 (15.54)  -0.01 | **0.0007** |
| TG (mg/dL) | (n = 19)  88.31 (34.71)  -0.19 | (n = 142)  96.56 (40.31)  -0.09 | (n = 140)  109.96 (49.35)  +0.10 | (n = 120)  104.35 (50.82)  +0.02 | 0.05^14^ |
| LDL-C (mg/dL) | (n = 17)  119.03 (38.14)  +0.04 | (n = 142)  122.33 (41.92)  **+0.09** | (n = 140)  111.42 (41.54)  **-0.08** | (n = 120)  114.88 (38.72)  -0.02 | 0.14^15^ |
| Vitamin B_12_ (ng/mL) | (n = 11)  341.21 (149.47)  -0.05 | (n = 75)  345.22 (214.33)  -0.04 | (n = 74)  378.36 (178.99)  +0.03 | (n = 64)  372.89 (246.62)  +0.02 | 0.76^16^ |
| Vitamin B_9_ (ng/mL) | (n = 11)  5.07 (1.41)  -0.19 | (n = 72)  7.50 (5.27)  +0.05 | (n = 73)  7.30 (4.60)  +0.03 | (n = 63)  6.24 (4.63)  -0.06 | 0.22^17^ |
| Vitamin D (ng/mL) | (n = 8)  19.35 (10.02)  -0.16 | (n = 103)  28.17 (16.80)  +0.02 | (n = 87)  28.53 (15.62)  +0.03 | (n = 90)  26.51 (16.84)  -0.03 | 0.42^18^ |
| PTH (pg/mL) | (n = 3)  52.14 (10.80)  -0.07 | (n = 57)  61.94 (30.56)  -0.10 | (n = 55)  80.78 (55.53)  +0.06 | (n = 38)  82.20 (57.86)  +0.06 | 0.09^19^ |
| FBG (mg/dL) | (n = 7)  365.57 (90.97)  -0.05 | (n = 48)  380.60 (109.39)  +0.01 | (n = 54)  384.28 (83.35)  +0.03 | (n = 51)  371.71 (84.77)  -0.03 | 0.89^20^ |

**Table note:**

Data are presented as mean (SD) and z-scores. Variables marked with an asterisk (*) were included in the Principal Component Analysis (PCA). Post-hoc comparisons between clusters were performed for selected variables using ANOVA test , with Bonferroni correction for multiple testing; p-values < 0.05 were considered statistically significant.

Pairwise p-values are indicated below:

1 I vs II = 0.19; I vs III = <0.0001; I vs IV = 0.0004; II vs III = <0.0001; II vs IV = 0.0005; III vs IV = 0.0009

2 I vs II = <0.0001; I vs III = <0.0001; I vs IV = <0.0001; II vs III = <0.0001; II vs IV = <0.0001; III vs IV = 0.59

3 I vs II = <0.0001; I vs III = <0.0001; I vs IV = <0.0001; II vs III = <0.0001; II vs IV = <0.0001; III vs IV = 0.003

4 I vs II = <0.0001; I vs III = <0.0001; I vs IV = <0.0001; II vs III = <0.0001; II vs IV = <0.0001; III vs IV = 0.96

5 I vs II = 0.020; I vs III = <0.0001; I vs IV = 0.0006; II vs III = <0.0001; II vs IV = 0.19; III vs IV = 0.07

6 I vs II = <0.0001; I vs III = <0.0001; I vs IV = <0.0001; II vs III = <0.0001; II vs IV = <0.0001; III vs IV = 0.50
7 I vs II = 0.0029; I vs III = <0.0001; I vs IV = <0.0001; II vs III = <0.0001; II vs IV = <0.0001; III vs IV = 0.53

8 I vs II = <0.0001; I vs III = <0.0001; I vs IV = <0.0001; II vs III = <0.0001; II vs IV = 0.15; III vs IV = <0.0001

9 I vs II = 0.77; I vs III = 0.99; I vs IV = 0.49; II vs III = 0.81; II vs IV = 0.36; III vs IV = 0.45

10 I vs II = 0.36; I vs III = 0.77; I vs IV = 0.81; II vs III = 0.99; II vs IV = 0.45; III vs IV = 0.49

11 I vs II = 0.87; I vs III = 0.83; I vs IV = 0.90; II vs III = 0.99; II vs IV = 0.99; III vs IV = 0.98

12 I vs II = 0.98; I vs III = 0.99; I vs IV = 0.91; II vs III = 0.79; II vs IV = 0.08; III vs IV = 0.46

13 I vs II = 0.79; I vs III = 0.0066; I vs IV = 0.0022; II vs III = 0.34; II vs IV = 0.48; III vs IV = 0.99

14 I vs II = 0.88; I vs III = 0.22; I vs IV = 0.50; II vs III = 0.07; II vs IV = 0.53; III vs IV = 0.76

15 I vs II = 0.99; I vs III = 0.88; I vs IV = 0.98; II vs III = 0.10; II vs IV = 0.44; III vs IV = 0.90

16 I vs II = 0.99; I vs III = 0.95; I vs IV = 0.96; II vs III = 0.77; II vs IV = 0.86; III vs IV = 0.99

17 I vs II = 0.39; I vs III = 0.47; I vs IV = 0.88; II vs III = 0.99; II vs IV = 0.41; III vs IV = 0.56

18 I vs II = 0.45; I vs III = 0.42; I vs IV = 0.63; II vs III = 0.99; II vs IV = 0.89; III vs IV = 0.84

19 I vs II = 0.98; I vs III = 0.74; I vs IV = 0.16; II vs III = 0.72; II vs IV = 0.18; III vs IV = 0.99

20 I vs II = 0.98; I vs III = 0.96; I vs IV = 0.99; II vs III = 0.99; II vs IV = 0.96; III vs IV = 0.89

Abbreviations: **ADLs**: Activities of Daily Living; **BUN:** Blood Urea Nitrogen; **BMI**: Body Mass Index; **CGA**: Comprehensive Geriatric Assessment; **CIRS**: Cumulative Illness Rating Scale; **CIRS ISC**, Cumulative Illness Rating Scale Severity Index; **CKD**: Chronic Kidney Disease Epidemiology Collaboration; **e-GFR:** Estimated Glomerular Filtration Rate; **ESS**: Exton-Smith Scale; **ENT:** Ear, Nose, and Throat; **FBG:** Fibrinogen; **GDS**: Geriatric Depression Scale; **Hb:** Hemoglobin; **HDL-C**: High-density lipoprotein Cholesterol; **IADLs**: Instrumental Activities of Daily Living; **LDL-C:** Low-Density Lipoprotein Cholesterol; **MMSE**: Mini-Mental State Examination; **MNA** Mini Nutritional Assessment; **PLTs:** Platelets Count; **POMA**: Tinetti Performance-Oriented Mobility Assessment; **PPT:** Performance-Based Physical Test*;* **PTH**: Parathyroid hormone; **RBCs**: Red Blood Cells; **SD**: Standard Deviation; **TG**: triglycerides; **WBCs:** White blood cells
